# Founder mutation in *KCNJ10* in Pakistani patients with EAST syndrome

**DOI:** 10.1002/mgg3.227

**Published:** 2016-06-07

**Authors:** Ola Abdelhadi, Daniela Iancu, Mehmet Tekman, Horia Stanescu, Detlef Bockenhauer, Robert Kleta

**Affiliations:** ^1^ Centre for NephrologyUniversity College LondonLondonUK

**Keywords:** Ataxia, epilepsy, kidney, Kir4.1, potassium channel, tubulopathy

## Abstract

**Background:**

EAST syndrome is an autosomal recessive disorder caused by loss‐of‐function mutations in the gene *KCNJ10*. Among the 14 pathogenic mutations described so far, the p.R65P mutation stands out as the most frequent one and is particularly associated with patients of Pakistani origin. As a result we aimed to establish the existence of a potential founder effect in the Pakistani population.

**Methods:**

To this end, we genotyped 12 patients from seven families and we compared disease haplotypes with ethnically matched control chromosomes. This haplotype was used together with demographic data for Pakistan to estimate the age of this founder mutation.

**Results:**

We identified a small homozygous 0.694 Mb region around the *KCNJ10* p.R65P mutation that had identical haplotypes in all of the patients which were completely absent in the control sample. Based on current demographic data and knowledge about disease frequency, we estimate that this particular p.R65P mutation arose 20 generations (about 500 years) ago.

**Conclusion:**

By knowing the prevalent mutation in a given population more efficient diagnostics can be performed and the families can benefit from specific counseling.

## Introduction

EAST syndrome (OMIM #612780) is an autosomal recessive disorder characterized by the combination of *E*pilepsy, *A*taxia, *S*ensorineural deafness, and renal salt‐wasting *T*ubulopathy (Bockenhauer et al. 2009; Scholl et al. 2009). The disease is caused by recessive mutations in the gene *KCNJ10* (also known as inwardly rectifying potassium channel, subfamily J, member 10 or Kir4.1) (Bockenhauer et al. 2009; Scholl et al. 2009, 2012; Reichold et al. 2010; Freudenthal et al. 2011; Cross et al. 2013; Parrock et al. 2013), expressed in the kidney, central nervous system, ear, and eye (Takumi et al. 1995; Ito et al. 1996; Kofuji et al. 2000; Marcus et al. 2002; Tanemoto et al. 2004; Bockenhauer et al. 2009). In the kidney, *KCNJ10* is located in the distal nephron's basolateral membrane where the dysfunction of this potassium channel leads to impaired salt reabsorption (Bockenhauer et al. 2009; Scholl et al. 2009; Reichold et al. 2010). The spectrum and severity of symptoms vary across identified EAST cases as well as among the members of the same family (Cross et al. 2013).

Fourteen different pathogenic mutations have been reported so far in *KCNJ10*. The missense mutation c.194G>C, replacing arginine with proline at the position 65 in the protein structure (p.R65P), is the most frequent mutation reported so far (Bockenhauer et al. 2009; Scholl et al. 2009, 2012; Reichold et al. 2010; Freudenthal et al. 2011; Cross et al. 2013; Parrock et al. 2013). The homozygous *KCNJ10* p.R65P mutation was first described in four Pakistani patients from a consanguineous family and subsequently has been found in several other families sharing the same ethnic background (Bockenhauer et al. 2009). In one case with mixed ethnic background, the p.R65P mutation has been reported in a compound heterozygous state, in association with a nonsense mutation (Scholl et al. 2009; Reichold et al. 2010). The arginine in position 65 of the *KCNJ10* protein is located in the first transmembrane motif of the channel; it is highly conserved and essential for its transport function, as shown by consistent experimental evidence (Bockenhauer et al. 2009; Reichold et al. 2010; Sala‐Rabanal et al. 2010; Tang et al. 2010; Williams et al. 2010; Zdebik et al. 2013; Chen and Zhao 2014). The mutation causes the loss of >80% of protein's transport activity (Sala‐Rabanal et al. 2010; Bandulik et al. 2011; Freudenthal et al. 2011). The very same amino acid is affected by another substitution, replacing the arginine with a cysteine, in an Algerian family with EAST syndrome (Freudenthal et al. 2011).

Knowing the prevalent mutation in a population has a significant impact on efficient diagnosis and specific counseling of affected families. As all the Pakistani patients investigated so far by our group are homozygous for p.R65P mutation, we considered the possibility of a founder effect in this population and we set to estimate its age. To our knowledge, no founder effect has been reported so far in EAST syndrome patients.

## Methods

### Patients

We utilized DNA samples from 12 EAST syndrome patients, six females and six males, with previously confirmed homozygous *KCNJ10* (GenBank reference sequence and version number: NG_016411.1; Gene ID: 3766) p.R65P mutation, and from four of their parents. These patients came from seven families and were the outcome of first cousin marriages of parents from Pakistani heritage. Two of the seven families were known also to be distantly related. Genetic studies were approved by the Institute of Child Health–Great Ormond Street Hospital Research Ethics Committee and the parents provided written informed consent.

### Whole genome SNP genotyping

The samples were genotyped using the Infinium HumanCore‐24 Beadchip, on an Illumina (San Diego, CA) platform, according to the manufacturer's recommendations. The genotype data were subjected to quality controls, which included filtering noninformative markers, genotype errors, and biased markers according to extreme minor allele frequencies for the Pakistani population.

### Haplotype reconstruction

Whole genome haplotype analysis was performed using Allegro as described previously (Bockenhauer et al. 2009). The individual alleles corresponding to the SNPs located on the long arm of chromosome 1, in the *KCNJ10* region, were imported into an Excel spreadsheet, and manual detection of a common disease haplotype for each EAST case was done by looking at shared arrangements of alleles among cases. By visual comparison, we determined the longest region of homozygosity shared by all the patients. Genetic position of the individual markers used in the analysis was obtained from The Human Genome Working Draft (http://genome.ucsc.edu/) and genetic distances calculated based on the estimation that 1 Mbp = 1 cM (Kent et al. 2002). We obtained haplotypes for 96 controls as well as the allelic frequencies for the Pakistani population from The 1000 Genome Project (www.1000genomes.org). We selected data corresponding to the Punjabi population as this is the main population in Pakistan, and therefore representative for our study (Taus‐Bolstad 2003; International Organisation for Migration, 2006).

### Statistical analysis

We compared the differences in allele frequencies between mutant chromosomes and normal ethnically matched chromosomes for the single markers within the disease haplotype as well as for the whole haplotype using a chi‐square test or Fisher's exact test, using Stata Statistical Software, release 12 (StataCorp, 2011). In both cases, we used a statistical threshold of 0.05. The control genotypes obtained from the 1000 Genome Project were tested manually to ensure no departure from Hardy–Weinberg equilibrium using chi‐square analysis and a statistical threshold of 0.05.

### Estimation of the age of the *KCNJ10* p.R65P mutation

To estimate the age of this mutation, we used DMLE+ v.2.3 software, a Bayesian linkage disequilibrium gene mapping method that relies on linkage disequilibrium between pathogenic mutations and multiple linked markers in patients and unrelated healthy controls (Reeve and Rannala 2002). We estimated the Pakistani population growth rate (*r*) using the formula N1 = N0e^(gr)^, where “N1” stands for the estimated population size today, “N0” is the estimated initial population size, and “g” the number of generations between the two time points (Vandermeer 2010). We estimated the disease allele frequency in Pakistani population based on a disease incidence of 1/100,000. We calculated the proportion of the population sampled (f) as described by Rannala and Slatkin (1998).

## Results

### Haplotype analysis

Haplotype analysis demonstrated a stretch of homozygosity of 0.694 Mb, encompassing four SNPs, rs4282816, rs2808661, rs6656979, and rs11577096, adjacent to the *KCNJ10* gene (Table 1). This haplotype was identical in all patients and could be identified in heterozygous state in the four genotyped parents. However, no match could be found for this haplotype in the control sample. Taking the flanking SNP markers into account identified a linked locus containing six SNPs in total, rs2852727, rs4282816, rs2808661, rs6656979, rs11577096, and rs7521304, covering an area of 1.255 Mb.

**Table 1:** Genetic position from the six SNPs and *KCNJ10* p.R65P mutation for the observed founder haplotype

SNP/mutation	Genetic distance from rs2852727 in Morgans (M)
rs2852727^a^	0.0
rs4282816	0.002059
rs2808661	0.004713
rs6656979	0.006952
rs11577096	0.009000
p.R65P	**0.009554**
rs7521304^a^	0.012550

a Flanking markers.

*KCNJ10 p.R65P* mutation is given in bold (RefSeqGene NG_016411.1).

The haplotypes covering the same markers and the same genetic distance were obtained from the 1000 Genomes data and compared with the disease haplotype observed both in patients and their relatives (Table 2). The 192 chromosomes in the control set (96 samples) had a total of 12 different configurations for the same block of SNPs shared by the patients, none of which were identical to the four SNP regions identified in our EAST syndrome cases. The 96 ethnically matched controls were found to be in Hardy–Weinberg equilibrium, thus confirming their suitability for our study.

**Table 2:** Frequency of alleles within the founder haplotype for *KCNJ10* p.R65P chromosomes and control chromosomes

Marker	Allele	Study group				*P* value^c^
		Patients (24 chromosomes)		1000 Genomes PJL controls^b^ (192 chromosomes)		
		*N*	%	*N*	%	
rs2852727	G^a^	23	96	105	55	0.0001
	A	1	4	87	45	
rs4282816	G^a^	24	100	75	39	<0.0001
	A	0	0	117	61	
rs2808661	A^a^	0	0	57	30	0.0008
	G	24	100	135	70	
rs6656979	C^a^	0	0	167	87	<0.0001
	A	24	100	25	13	
rs11577096	C^a^	0	0	172	90	<0.0001
	T	24	100	20	10	
rs7521304	T^a^	1	4	49	26	0.0194
	G	23	96	143	74	

a Reference allele. Alleles highlighted in gray correspond to the founder disease haplotype (*KCNJ10* RefSeqGene NG_016411.1).

b PJL controls (Punjabis in Lahore), Pakistan, sequenced as part of the latest phase three variant set of The 1000 Genome Project.

c Two‐tailed *P* value obtained from Fisher's exact or chi‐square analysis for one degree of freedom. Statistical threshold of 0.05 used. All *P* values obtained were statistically significant.

A Fisher's exact test of the proposed common disease haplotype in cases and controls produced a two‐tailed *P* value of less than 0.0001. Therefore, there is a statistically significant difference in the frequency of the common haplotype in cases versus controls. This difference was confirmed when we compared the allele frequency of the six individual markers within the analyzed haplotype as the frequencies were significantly different in mutant chromosome compared to control chromosomes.

### Estimating the age of p.R65P mutation

**Figure 1:**
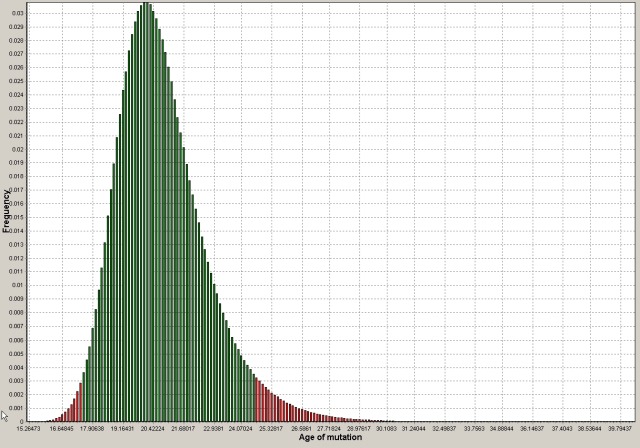
Age of p.R65P mutation estimated by DMLE+ v.2.3 software based on the founder haplotype. Green bars delimit the 95% confidence interval. *x*‐axis: age of mutation in generations (one generation = 25 years). *y*‐axis: frequency for each estimation out of a total of 10,000,000 iterations. The highest frequency was obtained for about 20 generations. The 95% confidence interval stretches from 17 to 25 generations.

We estimated that the *KCNJ10* p.R65P founder mutation has been introduced into the Pakistani population around 20 generations ago (rounded to the closest generation) with a 95% confidence interval (CI) between 17 and 25 generations. With each generation set to last 25 years, this means that the mutation probably appeared 500 years ago (95% CI: 425–625 years ago). Figure 1 shows a graphical representation of the results obtained from DMLE+ software with the histogram bars within the 95% confidence interval colored in green.

The demographic parameters were calculated as described in the Methods section and had the following values: a population growth rate of 0.68/generation for a current Pakistani population size of 190,337,989 (www.pwd.punjab.gov.pk) and a proportion of population sampled of 0.00002. Based on an estimated incidence of 1 in 100,000 EAST syndrome cases in Pakistan, we could calculate the approximate values for the disease prevalence and carrier frequency. Assuming that all the cases in the Pakistani population are due to *KCNJ10* p.R65P mutation, we inferred the frequency of the mutant allele as being 0.316%, and the carrier frequency as high as 1 (0.63%) in 159.

## Discussion

This study provides evidence that the EAST syndrome caused by *KCNJ10* p.R65P mutation in Pakistani patients is likely to be the result of a founder mutation that occurred around 500 years ago. So far this is the only mutation described in Pakistani patients with EAST syndrome.

Different missense, frameshift, and nonsense mutations have been identified so far in *KCNJ10* gene, in patients of different ethnicities (Bockenhauer et al. 2009; Scholl et al. 2009, 2012; Freudenthal et al. 2011; Thompson et al. 2011). Most mutations are found in one or a few families. However, all our patients of Pakistani descent are homozygous for the missense mutation p.R65P. In order to prove the founder effect we genotyped 12 patients and four parents from seven British Pakistani families and we compared the haplotypes containing the *KCNJ10* p.R65P mutation. The same haplotype was present in homozygous state in patients and heterozygous state in the four parents, carriers for the disease. This haplotype was absent in the control chromosomes. Given the high rate of consanguinity in Pakistani population, we considered the possibility that all the seven families might be related. However, except for two of them being distantly related, the other families had no identifiable connection, strengthening the idea of a founder mutation. The *KCNJ10* p.R65P mutation has not been found in patients from other ethnic background, further supporting the hypothesis of a founder effect within the Pakistani population.

The EAST syndrome is relatively recently described and as rare disease might be still an underdiagnosed disease. This is why we used in our study an estimated value of 1/100,000 for the disease incidence (Rannala and Slatkin 1998). If this estimation is close to the truth, EAST syndrome might be underdiagnosed in this population in Pakistan.

Mutation age calculation depends on a series of events like demographic changes, disappearance or recurrence of the same mutation in different, unrelated individuals, selection, or recombination (Slatkin and Rannala 2000; Rosenberg and Nordborg 2002). The algorithm included in DMLE+ program takes all these factors into account and the result is given in a range of confidence that includes all these variations (Rannala and Slatkin 1998; Reeve and Rannala 2002).

The demographic parameters have a significant impact on mutation age determination, and for this reason we chose more precise data, even if more recent, for our calculations. Pakistan, as a country has a relatively short history, but it originates in one of the oldest ancestral populations the Indus Valley Civilization, having a reported estimated population size of 5,000,000 people at its peak between 2500 and 1700 BCE (Kahn 2005). The multiple migrations and redistributions in the area might have had an impact both on the distribution of the mutation and the actual growth rate over a longer time. Since this ancient population seems to have founded most of the populations of the Indian subcontinent, including Pakistan, India, and Afghanistan, we chose to use more precise data given by the first Pakistani census, in 1951, to calculate the population growth rate (Kahn 2005). However, given the fact that these data are only 2.5 generations old, we consider the fact that the result might not reflect the complete evolution of the population. The same limitations have been met by other studies attempting to investigate founder mutations within Pakistani populations (Shaiq et al. 2012). Pakistan was part of the British Indian Empire and gained independence in 1947. This period of dissolution of the British Indian Empire witnessed the largest mass migration in history with millions of Muslims living in India relocating to Pakistan and millions of Hindus residing in Pakistan moving to India (Haub and Sharma 2006). In our experience, only Pakistani patients presented p.R65P mutation, supporting separation of this population from the other populations in South East Asia for the purpose of this study. Considering all these variations, we estimated that the *KCNJ10* p.R65P mutation occurred about 500 years or 20 generations ago (17–25 generations confidence interval). Around 1550 AD corresponds to the early times of Mughal Empire, ruled by a Turco‐Mongol dynasty and covering the actual territories of Pakistan, Afghanistan, India, and Bangladesh. Lahore, the capital of the Punjab Province of Pakistan, was for a short time the capital of Mughal Empire in the 16th and early 17th centuries.

## Conclusions

The presence of the *KCNJ10* p.R65P mutation at high frequency in patients of Pakistani descent only, associated with an identical haplotype including the disease allele in all affected individuals investigated by our team, strongly supports the hypothesis of a founder effect within this population. The origin of this mutation has been estimated to have arisen around 500 years ago, before the latest major migrations in the area. Knowledge about the genetics of EAST syndrome and the prevalent mutation p.R65P in this population supports targeted molecular diagnosis and enables provision of appropriate genetic counseling, particularly in a new disease like EAST syndrome.

## Conflict of Interest

None declared.
